# A parametric metamodel of the vehicle frontal structure accounting for material properties and strain-rate effect: application to full frontal rigid barrier crash test

**DOI:** 10.1016/j.heliyon.2022.e12397

**Published:** 2022-12-13

**Authors:** Angelo Pasquale, Victor Champaney, Youngtae Kim, Nicolas Hascoët, Amine Ammar, Francisco Chinesta

**Affiliations:** a ESI Group Chair @ PIMM Lab, ENSAM Institute of Technology, 151 Boulevard de l'Hôpital, F-75013, Paris, France; b Hyundai Motor Group, Virtual Technology Innovation Research Lab, Automotive R&D Division, 150 HyundaiYeonguso-ro, Namyang-eup, Hwaseong-si, Gyeonggi-do, 18280, Republic of Korea; c ESI Group Chair @ LAMPA Lab, ENSAM Institute of Technology, 2 Boulevard du Ronceray BP 93525, 49035 Angers cedex 01, France; d ESI Group, Parc Icade, Immeuble le Seville, 3 bis, Saarinen, 94528, Rungis Cedex, France

**Keywords:** MOR, sPGD, Multi-sPGD, PODI, Curves metamodeling, Vehicle frontal structure, Crashworthiness, Lightweightness, Parametric strain-hardening laws

## Abstract

In the automotive industry, building parametric surrogate models is a fundamental tool to evaluate, in real time, the performance of newly designed car components. Such models allow to compute any Quantity of Interest —QoI—, such as a specific safety protocol index, for any choice of material and/or geometrical parameters characterizing the component, within the stringent real time constraint. For instance, they can be exploited to guarantee safer designs (e.g., maximizing energy absorption by the crash boxes) or to reduce manufacturing costs (e.g., minimizing the mass of a specific structure under some safety protocol constraints). In general, these parametric simulation tools allow a significant gain in terms of manufacturing costs and time delays during the investigation phase. In this study, we focus on the vehicle frontal structure system considering its performance in a full-frontal crash scenario. In the front structure system we parameterize the crash boxes (left and right) and the inner/outer side front members (left and right, front and rear) with respect to the part thickness and the material parameters characterizing the Krupkowski plasticity curve. Moreover, Strain Rate Effect is also taken into account via Neural Network based regressions, whose training dataset comes from experimental data. The parametric metamodel is built via Non-Intrusive PGD —NI–PGD— strategies, relying on a sparse sampling of the parametric space, and allowing a quite reduced number of High Fidelity —HiFi— simulations. A novel strategy based on clustering and classification, known as Multi-PGD, is also applied and numerically verified.

## 1 Introduction

In this paper, we revisit some state-of-the-art Model Order Reduction —MOR— technologies and propose new advances to address safety analyses in the automotive industry.^2^ The main goal of the work is to build a parametric metamodel of the vehicle frontal structure (involving material properties and design parameters), enabling accurate real-time evaluations of its response to a crash scenario. Such model allows fast optimization, inverse analysis and uncertainty propagation; it can be exploited, for instance, to guarantee safer designs (e.g., maximizing energy absorption by the crash boxes) or to reduce manufacturing costs (e.g., minimizing the mass of a specific structure under some safety protocol constraints).

MOR is a really wide topic, largely covered in the existing literature [1], [2], [3]. This work primarily focuses on snapshots-based reduced-order modeling (a subject that is extensively discussed in [2]) and, particularly, on the Proper Orthogonal Decomposition —POD— and on the sparse Proper Generalized Decomposition —sPGD—. Extensive reviews and investigations on such techniques can be found in [4], [5], [6], [7], [8], [9], [10], [11], [12]. In particular, in the recent work [10], the authors deal with parametric metamodeling of curves, based on POD, PGD, data alignment, data clustering and data classification, with emphasis in computational materials science. Moreover, in [10], authors exploit the surrogates to quantify and propagate uncertainty, obtaining parametric statistical bounds for the predicted curves. This paper follows and further develops the methods introduced in [10], focusing in particular on the study-case of vehicle crash simulations. Within such context, even though similar workflows have already been applied in literature (e.g., [13], [14], [15]), this work presents elements of novelty both from the viewpoint of the applied methodologies and from the analyzed model features. In terms of methodologies, the multi-sPGD is applied, combining the sPGD regression with clustering and classification algorithms based on crash modes. The main motivation beyond the choice of the method is that the sPGD allows the usage of a reduced number of offline simulations (which are highly expensive computationally when the full vehicle is considered). This is not trivial when working in high-dimensional spaces and with highly non-linear problems, which is the case studied in this work. Moreover, the combination of the sPGD with clustering and classification is an innovative sub-modeling procedure allowing to improve the model accuracy. In terms of model features, we focus on the vehicle frontal structure, whose thicknesses and materials' Krupkowski strain-hardening laws are parametric (for a total number of 13 parameters). A standard sampling strategy based on Latin-Hypercube Sampling —LHS— to select the Krupkowski parameters would not have allowed physical-consistent results. For this reason, a specific sampling based on physical materials' properties has been employed, representing another innovative part of the work. Particularly, this makes use of the *k*-nearest neighbors algorithm (alternatives would be manifold learning techniques) to move close to the manifold of experimental data (existing and tested materials). Moreover, another point of novelty is the usage of Neural Networks (trained on experimental data) to account for strain-dependent plasticity in the metamodel.

The sPGD regression is employed following the methods widely detailed in Section 2 of [10]. More specifically, the approximation of curves is performed within a POD-based approach (Subsection 2.3.3.1 of [10]) and a quality enhancement of the regression is achieved through a clustering-classification strategy (Subsection 2.4 of [10]).

Since works such as [8], [10] extensively expose all the specifics of the methods, hereafter we only briefly go over the sPGD method's core concept.

For the sake of simplicity and without loss of generality, we consider function u(x,y), with *x* and *y* two parameters defined in $\Omega \subset R^{2}$. We look for the approximate $u^{M}(x,y)$ expressed in the separated form given in Eq. (1)

(1) $$u^{M}(x,y)=\sum_{m=1}^{M}X_{m}(x)\cdot Y_{m}(y)=\sum_{m=1}^{M}(N_{m}^{x^{T}}(x)a_{m}^{x})\cdot (N_{m}^{y^{T}}(y)a_{m}^{y}),$$

where *M* refers the number of terms (rank) of the finite sum decomposition, vectors $N_{m}^{x}(x)$ and $N_{m}^{y}(y)$ contain the functions involved in the approximation of $X_{m}(x)$ and $Y_{m}(y)$ respectively, and $a_{m}^{x}$ and $a_{m}^{y}$ contain the associated (searched) coefficients.

With the first M−1 modes already calculated, the obtention of the *M*-mode results from the minimization problem

$$(N_{M}^{x^{T}}(x)a_{M}^{x})\cdot (N_{M}^{y^{T}}(y)a_{M}^{y})=$$

(2) $$\underset{a_{M}^{x⁎},a_{M}^{y⁎}}{\mathrm{arg min}}\;\sum_{\begin{array}{c} i=1 \end{array}}^{D}{\left|u(x_{i},y_{i})-u^{M-1}(x_{i},y_{i})+(N_{M}^{x^{T}}(x_{i})a_{M}^{x⁎})\cdot (N_{M}^{y^{T}}(y_{i})a_{M}^{y⁎})\right|}^{2},$$

where *D* is the number of available data-points, $\left(x_{i},y_{i}\right)$, i=1,…,D, for which the solution is assumed known (high-fidelity solutions) $u(x_{i},y_{i})$.

Eq. (2) proceeds by calculating iteratively coefficients $a_{m}^{x}$ and $a_{m}^{y}$, from Eq. (3) and (4), respectively

(3) $${\left\|K_{M}^{x}a_{M}^{x}-r\right\|}^{2},$$

(4) $${\left\|K_{M}^{y}a_{M}^{y}-r\right\|}^{2},$$

both solved in a least square sense, with $K_{M}^{x}$, $K_{M}^{x}$ and **r** derived from Eq. (2).

To avoid overfitting the richness of the approximation bases used at each level (*m*) is controlled, by increasing gradually the approximation degree when increasing *m*.

The paper is structured as follows. In Section 2 the target system is presented, describing in detail the parametric vehicle frontal structure. Section 3 represents an introductory example to the sPGD based regression within the PODI MOR builder; only the thicknesses are considered in this case. Section 4 describes in detail the integration of material properties and strain-rate dependent Krupkowski plasticity into the sPGD model. Section 5 presents the multi-PGD approach based on clustering different crash dynamics. Section 4 and 5 represent the main original contributions of this work. Section 6 is a short conclusion on the proposed method and discusses several industrial applications.

## 2 Target system

**Figure 1:**
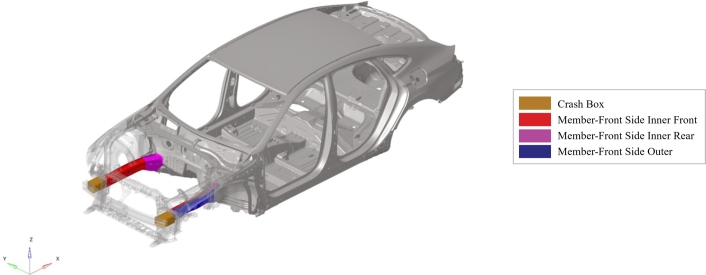
Full car model and highlighted target system.

**Figure 2:**
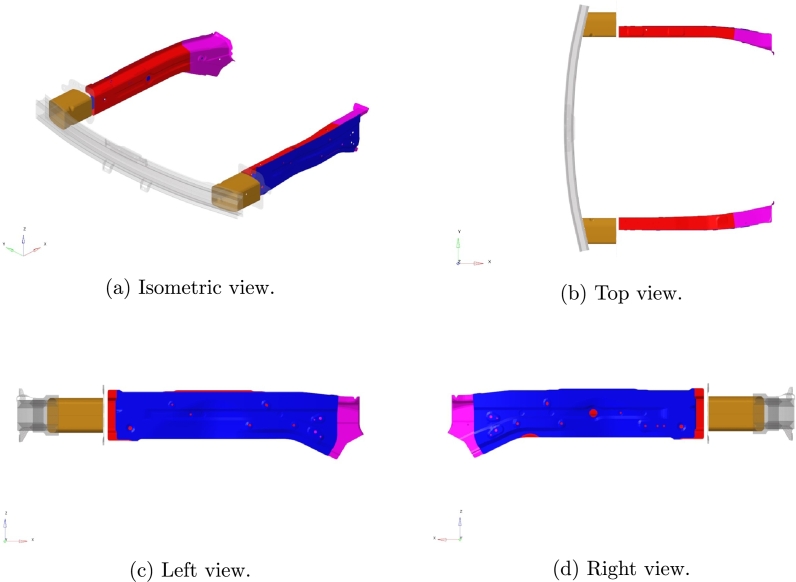
Zoom of the target system.

The vehicle front structure composed by the crash boxes, the front side inner members (front and rear) and the front side outer member is parametrized. The target system is shown in Fig. 1, while Fig. 2 gives multiple zoomed views of the parametric parts.

**Figure 3:**
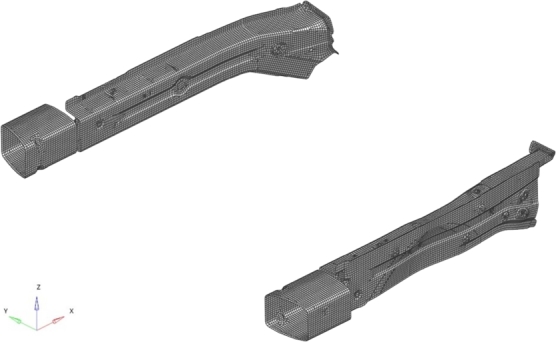
Mesh of the target system.

Numerical simulations of the vehicle full-frontal rigid barrier crash are carried-out using the finite element method with the commercial software LS-Dyna used by Hyundai. The frontal structure is discretized by means of 23233 shell elements (23737 nodes), as shown in Fig. 3, and using 5 integration points through-the-thickness. The mesh of the full vehicle consists of several millions of elements (solids, beam and thin shell types) and has been verified being fine enough to ensure accurate high-fidelity simulations. Moreover, we stress the fact that the quality of the parametric model is evaluated with respect to LS-Dyna simulations, and not with respect to the experimental tests of the full-car crash test. This because, as usually done in reduced-order modeling, the target is the solution we would obtain by performing the full-order simulation.

**Figure 4:**
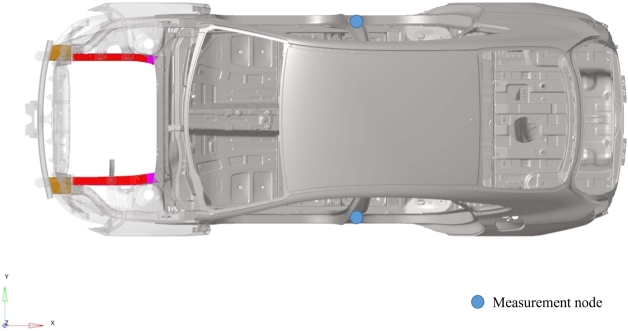
Location of measurement nodes (LH/RH).

Quantities of interest are extracted at the measurement nodes located at the bottom of the B-pillar (left and right), as shown in Fig. 4. Particularly, we focus on displacement, velocity and acceleration along the *x*-direction. The crash pulse curve (acceleration time history) is obtained by deriving the velocity signal computed by LS-Dyna and then filtering according to the CFC60 filter class (based on the iso6487 standard).

**Figure 5:**
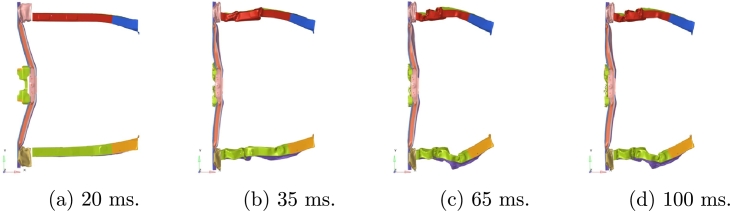
Deformation behavior evolving in time during crash.

The initial velocity of the vehicle is approximately 55 km/h and a full frontal rigid barrier crash is simulated over the first 100 ms. Acceleration is measured in standard gravity *g*. Fig. 5 shows the deformation behavior of the parts of interest.

Some important crash severity parameters are listed here below:

• The 1st peak of acceleration curve is defined as the maximum value of the filtered acceleration curve over the first 27 ms. The upper-bound of this safety index is a peak value of 25 g.
• The Occupant Load Criterion —OLC— which indicates the minimum occupant acceleration, induced by a given crash pulse under the protection of the ideal restraint system [16], [17], [18]. When the occupant reaches the distance of 65 mm, it is assumed that the occupant is optimally restrained. The EuroNCAP procedure for the computation of OLC is detailed in [19] and described here below:
  1. let $v_{0}$ and *v* denote *x*-velocity at initial time $t_{0}$ and a generic time *t*, respectively;
  2. let $S_{1}(t)=\int_{0}^{t}(v_{0}-v)dt$;
  3. find the time $t_{2}$ such that $S_{1}(t_{2})=65$ mm;
  4. define $S_{2}(t)=(t-t_{2})(v_{0}-v)/2$;
  5. find the time $t_{3}$ such that $S_{2}(t_{3})-S_{1}(t_{3})=300$ mm;
  6. use the definition $\mathrm{OLC}=-\frac{v(t_{3})-v(t_{2})}{t_{3}-t_{2}}$.
  Values in between 30 g and 35 g are considered as an acceptable crash performance in our study.
• The Ride-Down Energy —RDE— computed by taking the integral of the acceleration-displacement curve *a*-*x*. Detailed analysis for ride-down mechanism can be found in [20], [21], [22], [23]. The concept of ride-down efficient and the ride-down existent criteria are discussed in [20], [23]. Denoting with $E_{\text{rd}}$ the ride-down energy density and with *E* the initial occupant kinetic energy density (assuming for the occupant the same velocity as the impact speed), then the ride-down energy density rate is defined as $R_{\text{rd}}=E_{\text{rd}}/E$ and we consider the occupant injury to be acceptable for rate values under < 50%.
• The rebound time, that is the time instant in which the velocity curve gets to zero.

## 3 Parametric model with part thicknesses

**Figure 6:**
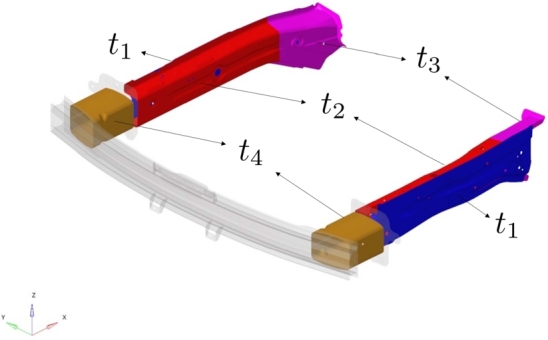
Sketch of parts with parametric thickness.

For the sake of methodological illustration, we first build a parametric model considering only the part thicknesses. Table 1 summarizes the original thickness of parts of interest as well as the parametric ranges covered by the model of the frontal structure, sketched in Fig. 6.

**Table 1:** Thickness information.

Part (LH/RH)	Original Thickness [mm]	Parameter Range [mm]
Member-Front Side Outer	1.4	*t*_1_∈ [0.8, 2.3]
Member-Front Side Inner Front	1.8	*t*_2_∈ [0.8, 2.3]
Member-Front Side Inner Rear	2	*t*_3_∈ [0.8, 2.3]
Crash Box	3.2	*t*_4_∈ [2, 4]

10 High-Fidelity simulations are chosen by means of a Latin Hypercube Sampling. The sampled points in the parametric space are reported in Table 2.

**Table 2:** DoE for thickness (data in mm).

Run	*t*_1_	*t*_2_	*t*_3_	*t*_4_
1	1.080	1.689	1.927	2.426
2	2.020	1.364	1.499	3.583
3	1.763	1.832	2.165	3.320
4	0.868	1.972	1.045	2.748
5	1.678	2.114	1.383	3.040
6	1.122	1.487	1.133	2.314
7	2.207	0.943	2.092	2.018
8	1.497	1.115	0.914	2.964
9	1.923	2.158	1.639	3.747
10	1.340	1.026	1.836	3.971

**Figure 7:**
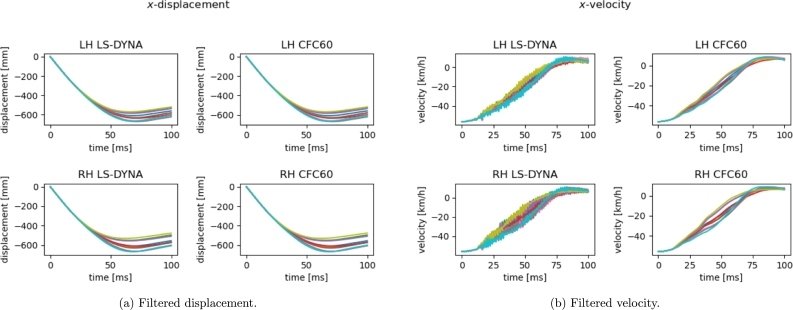
Filtered curves for 10 runs of Table 2 (LH and RH measurement points at top and bottom, respectively).

In Fig. 7, we give the plots of the displacement and velocity curves obtained by integration of the filtered acceleration. The sub-figures at top and bottom are related to left-hand (LH) and right-hand (RH) side measurement node, respectively (as shown in Fig. 4). Moreover the negative sign is related to the fixed reference system and chosen positive direction.

**Figure 8:**
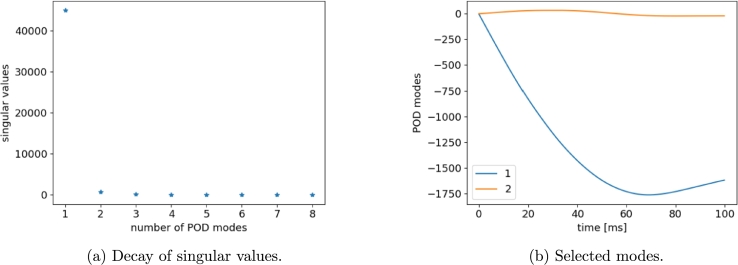
POD results for LH *x*-displacement snapshots.

**Figure 9:**
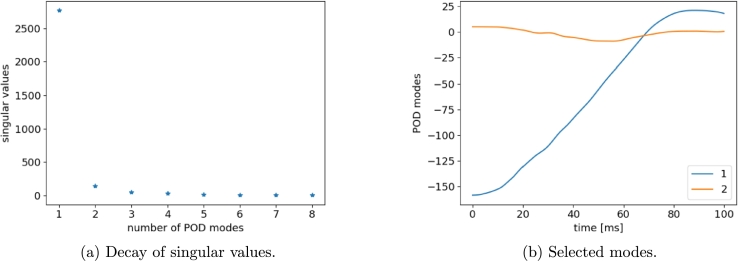
POD results for LH *x*-velocity snapshots.

Following the procedure described in Section 2 of [10], a POD basis is extracted from 8 Hi-Fi simulations, while remaining 2 (run 3 and run 8) are taken for testing. Analyzing the singular values pattern, we deduce that two modes contain the most information. For instance, Figure 8, Figure 9 show the POD results in the case of displacement and velocity computed at the left-side of the vehicle (LH), respectively. A basis of quite few time modes (two in this example) is thus retrieved employing the truncated POD and, then, the sPGD method is applied to predict the coefficients (weights) of such modes. Once the model has been trained and calibrated, new coefficients can be predicted for a novel choice of parameters and time functions are reconstructed using the reduced POD basis.

**Figure 10:**
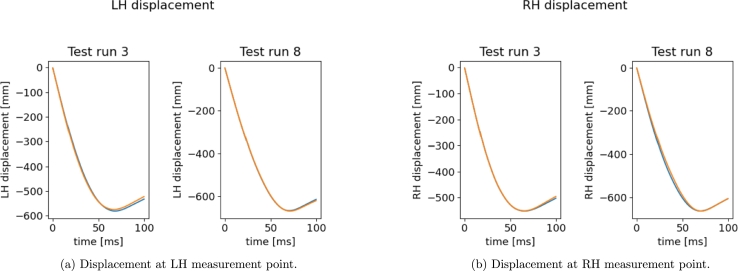
Reconstructed *x*-displacement using the sPGD models (orange: Hi-Fi simulation, blue: Reduced Model).

**Figure 11:**
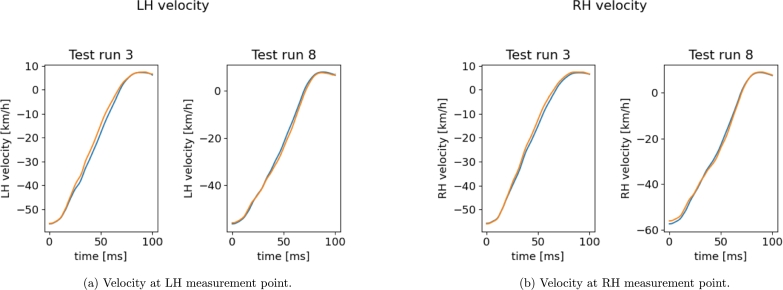
Reconstructed *x*-velocity using the sPGD models (orange: Hi-Fi simulation, blue: Reduced Model).

Figure 10, Figure 11 show the results on the test runs, for the displacement and velocity respectively. Both the results show great accuracy.

## 4 Accounting for material properties

The core of the work is considering material properties in the metamodel. With the aim of simulating virtual plastic materials, a Krupkowski strain-hardening law is considered, as usual in the high-fidelity models. For this to be done, two important novelties are introduced. The first one concerns the Design of Experiments —DoE— and, particularly, a physics-informed sampling strategy following the manifolds experimentally observed. The second one is related to the strain-dependent plasticity accounted by using Neural Networks, respecting the static and dynamic tests experimentally performed over materials specimens. These points are explained in detail hereafter.

### 4.1 Sampling strategy

In this study, the material properties of the front side members (steel parts) are also considered as problem parameters. For each part, we consider the 3 parameters $\left(n,K,\varepsilon_{0}\right)$ characterizing the Krupkowski strain-hardening law

$$\sigma =K{\left(\varepsilon +\varepsilon_{0}\right)}^{n},$$

linking the True Strength and the True Strain.

**Figure 12:**
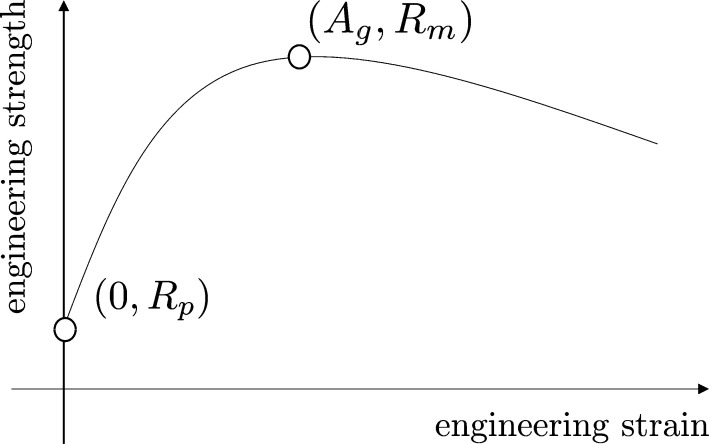
Plasticity curve and location of sampled points.

Since a Latin Hypercube Sampling —LHS— for the point $\left(n,K,\varepsilon_{0}\right)$ could lead to nonphysical results, we perform the sampling over three physical properties: the Yield Strength YS ($R_{p}$), the Ultimate Tensile Strength UTS ($R_{m}$) and the Uniform Elongation U-El ($A_{g}$, in %). Fig. 12 shows the location of such points over a typical plasticity curve linking the Engineering Strength and Engineering Strain.

From the sampled tuple (YS, UTS, U-El), we compute the corresponding Krupkowski parameters $\left(K,n,\varepsilon_{0}\right)$ by means of a non-linear optimization algorithm, detailed hereafter (Algorithm 1). Once such parameters are computed, the Krupkowski plasticity curve (ε,σ) identifies the material of a specific part.

**Algorithm 1:**
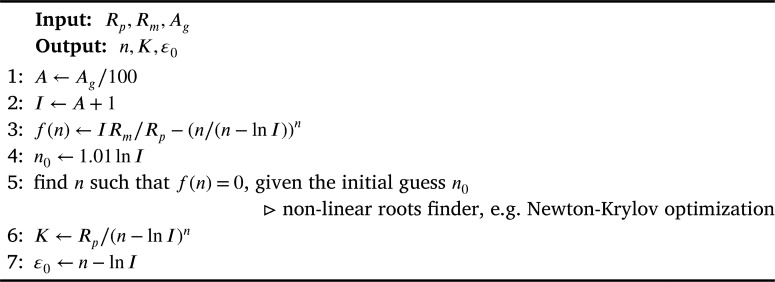
Computation of Krupkowski plasticity parameters from material properties

As one can deduce from Fig. 12, the sampling of material properties shall also meet some requirements to ensure realistic plastic behaviors. For instance, if values of $R_{m}$ and $R_{p}$ are too close, then the fitted curve would be meaningless (perfect plasticity). To avoid such uncomfortable configurations, a good alternative is based on sampling the Yield Ratio, $\text{YR}=\frac{\text{YS}}{\text{UTS}}=\frac{R_{p}}{R_{m}}$ and fix a suitable lower bound on this quantity.

### 4.2 Strain-rate effect

Since Strain-Rate Effect is considered in our simulations, the material of a part is identified by a rate-dependent plasticity curve (i.e., a plasticity curve for each rate). To correctly account for that in our parametric model, we exploit available experimental data collecting results of tests performed over specimens ranging from Mild Steel to Press Hardened Steel —PHS—. Such dataset links material properties observed at quasi-static test ${\left(\text{YS},\text{UTS},\text{U-El}\right)}^{\text{QS}}$ with the ones obtained at dynamic test under a strain rate *r*, ${\left(\text{YS},\text{UTS},\text{U-El}\right)}_{r}^{\text{D}}$. Each tuple of material properties identifies a plasticity curve, via Algorithm 1. For a given strain rate, we can therefore build a regression model whose features are the S-S curves at quasi-static test, while the targets are the corresponding S-S curves at dynamic test. Our data collect 1080 curves and account for 8 different strain rates (0.008,0.1,1,5,10,50,100,200) ${\text{s}}^{-1}$, that is 135 curves for a given strain rate. Data observed at strain rate equal to 0.008 represent the quasi-static test. For each rate, a Single-layer Fully Connected Neural Network is trained (using a ReLU activation function), with a ratio between train and test data is set as 9:1. Moreover, a standardization based on the usual Min-Max scaler is used to normalize the input features prior to model fitting.

Finally, for each part (Member-Front Side Outer, Member-Front Side Inner Front, Member-Front Side Inner Rear), the sampling procedure reads as follows:

1. sample ${\left(\text{UTS},\text{YR},\text{U-El}\right)}^{\text{QS}}$, i.e. at strain rate 0.008 ${\text{s}}^{-1}$;
2. calculate YS from YR: YS = UTS ⋅ YR;
3. for each rate *r* in (0.1,1,5,10,50,100,200) ${\text{s}}^{-1}$:
  (a) use the corresponding trained NN model to predict the tuple ${\left(\text{YS},\text{UTS},\text{U-El}\right)}_{r}^{\text{D}}$ accounting for strain rate;
  (b) from the sampled tuple ${\left(\text{YS},\text{UTS},\text{U-El}\right)}_{r}^{\text{D}}$, compute the corresponding plasticity parameters ${\left(n,K,\varepsilon_{0}\right)}_{r}$ via Algorithm 1 and, given the elongation *ε*, compute the curve ${\left(\varepsilon ,\sigma \right)}_{r}$.
4. generate the material collecting the rate-dependent plasticity curves ${\left(\varepsilon ,\sigma \right)}_{r}$.

For the sake of clarity, we underline that the Strain-Rate is not directly related to the Design of Experiments. After the sampling of a virtual material through its properties at quasi-static test, its response to Strain-Rate is predicted through the trained Neural-Networks models, allowing to obtain a plasticity curve for each rate characterizing such newly defined material.

**Figure 13:**
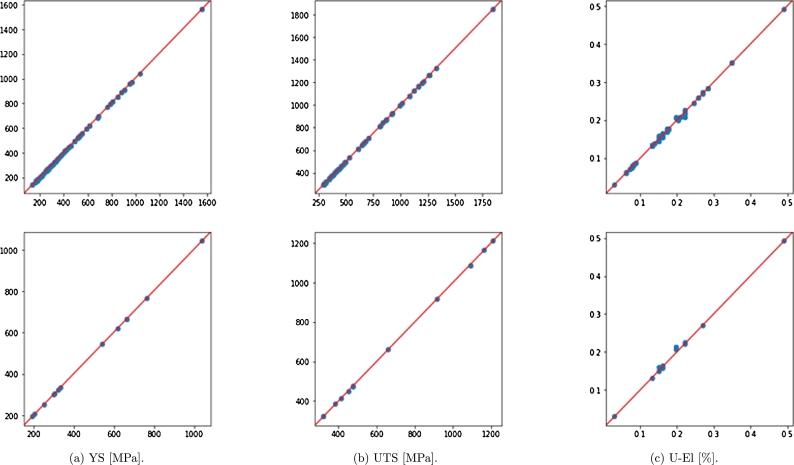
Predicted versus true values in case of rate 0.1; top: training data, bottom: test data.

Fig. 13 shows the results of NN predictions over train and test data, for Strain Rate 0.1. Table 3 gives the MSE values over train and test, which are small enough compared to the order of magnitude of the parameters.

**Table 3:** MSE values for Neural-Network predictions.

Strain Rate [s^−1^]	YS [MPa]		UTS [MPa]		U-El [%]	
	Train	Test	Train	Test	Train	Test
0.1	11.275	8.391	2.844	6.087	1.219 ⋅10^−5^	5.318 ⋅10^−5^
1	7.886	18.260	9.046	18.271	2.942 ⋅10^−5^	2.937 ⋅10^−5^
5	8.830	15.036	10.964	14.333	2.799 ⋅10^−5^	3.743 ⋅10^−5^
10	21.110	38.357	10.350	15.002	2.687 ⋅10^−5^	2.757 ⋅10^−5^
50	19.942	28.299	62.160	49.671	1.814 ⋅10^−5^	2.015 ⋅10^−5^
100	19.452	22.485	23.471	32.218	2.414 ⋅10^−5^	3.319 ⋅10^−5^
200	10.318	16.290	11.551	23.754	5.559 ⋅10^−6^	5.116 ⋅10^−6^

Since the Strain-Rate —SR— models are trained over experimental data, one needs to pay special attention to point 1. Indeed, if the sampled virtual material is too far from the ones used for training the Neural-Network models, we cannot expect reliable Strain-Rate curve predictions (point 3). A standard Latin Hypercube Sampling would definitely not be a good approach, since the experimental data belong to a small manifold inside the hypercube. The sampling strategy we use is thus based on a *k*-nearest neighbors —KNN— algorithm and summarized in Algorithm 2, which allows us to move close to the training dataset without having to know the manifold to which the data belong (otherwise, one could investigate the data distribution through specific manifold learning techniques).

**Algorithm 2:**
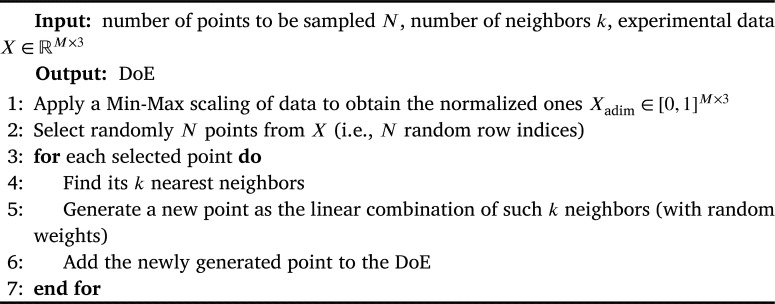
KNN-based sampling of material properties

### 4.3 Parametric metamodel construction

**Figure 14:**
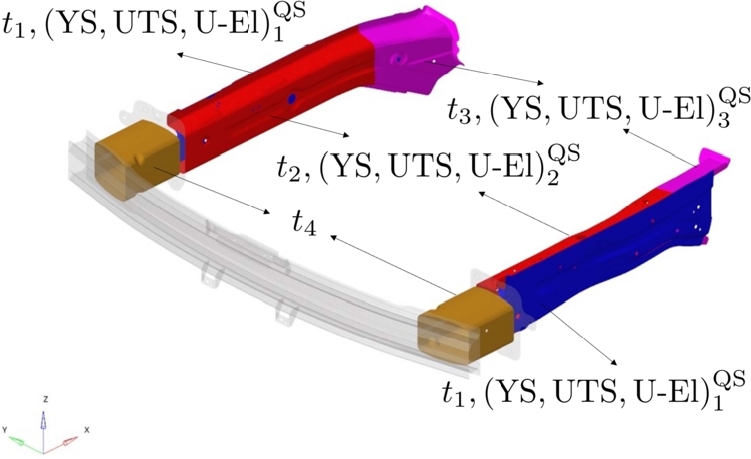
Sketch of parts with parametric thickness and material properties.

The new parametric model accounts for 13 parameters, as reported in Table 4 and sketched in Fig. 14. The three steel parts (Member-Front Side Outer, Member-Front Side Inner Front, Member-Front Side Inner Rear) have parametric material properties and thickness, while the Crash Boxes have only parametric thickness. 70 Hi-Fi simulations have been performed, 66 are taken for training the model while remaining 4 for validation.

**Table 4:** DoE structure for thickness *t* and material parameters ${\left(\text{YS},\text{UTS},\text{U-El}\right)}^{\text{QS}}$.

Outer		Inner Front		Inner Rear		Crash Box
*t*_1_	${\left(\text{YS},\text{UTS},\text{U-El}\right)}_{1}^{\text{QS}}$	*t*_2_	${\left(\text{YS},\text{UTS},\text{U-El}\right)}_{2}^{\text{QS}}$	*t*_3_	${\left(\text{YS},\text{UTS},\text{U-El}\right)}_{3}^{\text{QS}}$	*t*_4_

**Figure 15:**
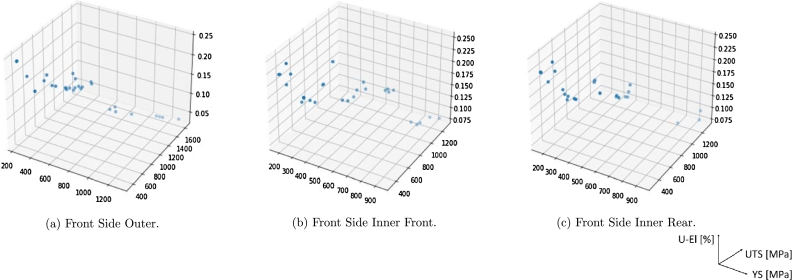
30 sampled points ${\left(\text{YS},\text{UTS},\text{U-El}\right)}^{\text{QS}}$ using the KNN-based algorithm.

The parameter range for thicknesses is the same as the introductory study of Section 3, reported in Table 1. Material properties at quasi-static test have been sampled via the KNN-based Algorithm 2 and, according to the available experimental data, they range from Mild Steel to Press Hardened Steel. Fig. 15 shows, for instance, 30 sampled points using this procedure, for the three parts. The material properties for one simulation are ${\text{MAT}}_{P}={\left(\text{YS},\text{UTS},\text{U-El}\right)}_{P}^{\text{QS}}$, with P=1,2,3 the corresponding part, meaning in total 9 material parameters. Such parameters are obtained taking the sampled point in the three 3D plots of Fig. 15 simultaneously. From the points sampled at quasi-static test, we can compute the parameters accounting for strain rate by means of the trained Neural-Network, as detailed in Subsection 4.2.

**Figure 16:**
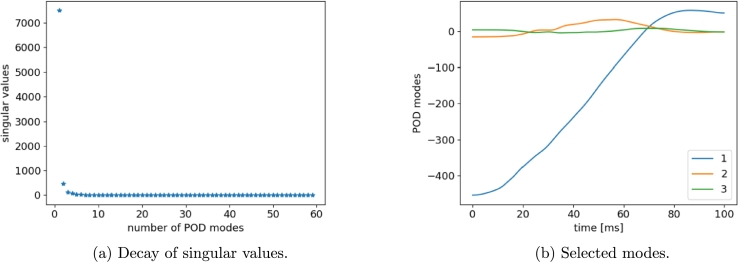
POD results for LH *x*-velocity snapshots.

Analyzing the singular values pattern, we deduce that three modes contain the most information. For instance, Fig. 16 shows the POD results in the case of velocity computed at the left-side of the vehicle (LH). The sPGD regression is thus applied as described in the previous section.

**Figure 17:**
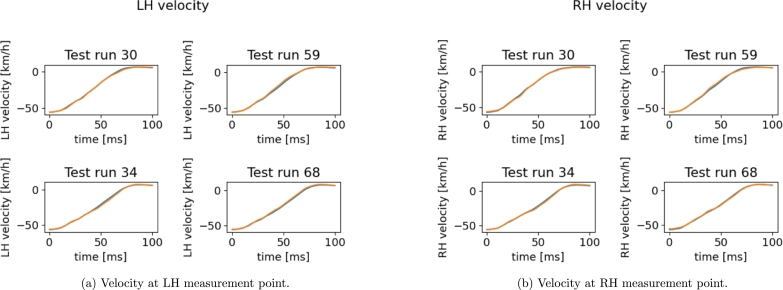
Reconstructed *x*-velocity using the sPGD models (orange: Hi-Fi simulation, blue: Reduced Model).

Fig. 17 shows the plots of predicted curves for testing points and Table 5 gives the related $L^{2}$ norm relative errors.

**Table 5:** *L*^2^ norm relative errors on test.

Run 30		Run 59		Run 34		Run 68	
LH	RH	LH	RH	LH	RH	LH	RH
0.023	0.023	0.036	0.033	0.014	0.029	0.033	0.023

**Figure 18:**
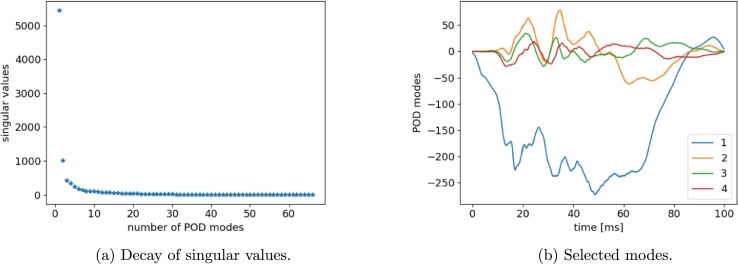
POD results for LH *x*-acceleration snapshots.

The same study is done over acceleration curves, for which first four modes are selected after a scaling analysis on singular values (see Fig. 18).

**Figure 19:**
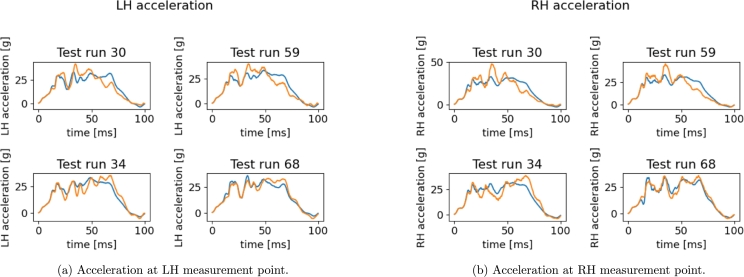
Reconstructed *x*-acceleration using the sPGD models (orange: Hi-Fi simulation, blue: Reduced Model).

In Fig. 19 predictions on acceleration curves are given. Regardless a well captured global shape, the oscillations and peaks amplitudes characterizing the transitory regimen are mostly wrong. One can notice that in runs 30 and 59 the measurement node starts decelerating at almost 45 ms. Different behavior is observed for runs 34 and 68 where the point keeps accelerating up to almost 65 ms and suddenly decelerates with a more pronounced slope. Such simulations highlight substantially different crash dynamics. The low accuracy of predictions can thus be ascribed to a model which is mixing and averaging such dynamics. These comments motivate the clustering-based approach investigated in the next section.

## 5 Multi-PGD

**Figure 20:**
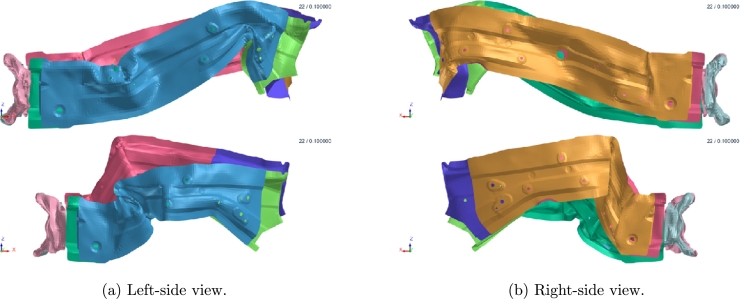
Two different crash modes, at bottom and top. Snapshots taken at the last timestep.

**Figure 21:**
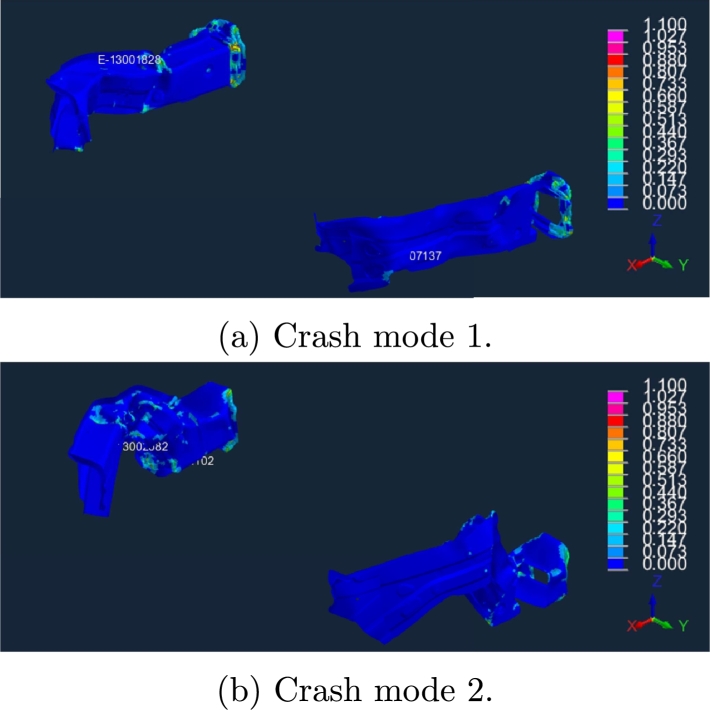
Two different crash modes (plastic-strain over the deformed structure with isometric view). Snapshots taken at the last timestep.

Further improvements can be reached through the multi-regression strategy briefly discussed in Section 2.4 of [10]. The idea is basically to cluster the high-fidelity simulations according to the most relevant crash mode, e.g. buckling and compression behaviors, as emphasized in Figure 20, Figure 21. Once a cluster criterion is established, separate reduced models are built. A classification algorithm is also necessary since, during the online phase, one needs to identify the right cluster for a new simulation (coming for a new choice of parameters).

### 5.1 Clustering

**Figure 22:**
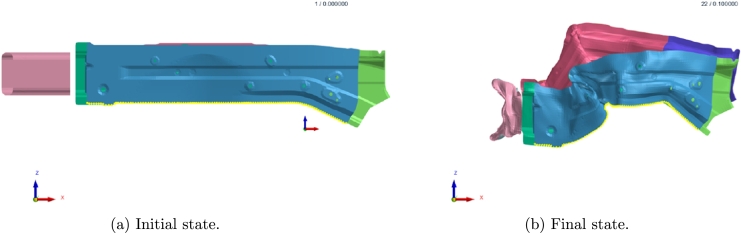
Left side member lower boundary displacement. Nodes of interest are highlighted in yellow.

The approach followed in this work is based on clustering the high-fidelity simulations according to the displacement of lower boundary nodes of the left side member as illustrated in Fig. 22. A similar clustering procedure was also applied in [14].

First, a reference deformation mode is computed for a specific configuration of parameters, reported in Table 6 (same layout as Table 4).

**Table 6:** Configuration of parameters for base mode definition.

Outer		Inner Front		Inner Rear		Crash Box
1.4	SPFC980Y	1.8	SPFC590DP	2	SPFC590DP	3.2

**Figure 23:**
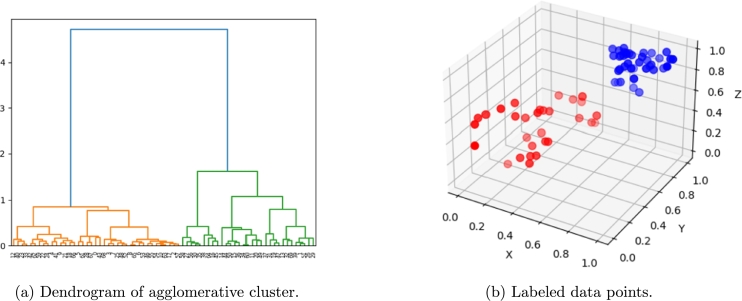
Hierarchical clustering results.

Then, for each simulation we compute the mean distance of its lower boundary nodes to such reference mode (each coordinate *x*, *y* and *z* separately) and use a min-max scaling to normalize data. In such a way, a unique point in three-dimensional space is associated to each simulation. A hierarchical cluster based on ward linkage and euclidean affinity is applied and two clusters are obtained, as shown in Fig. 23.

**Figure 24:**
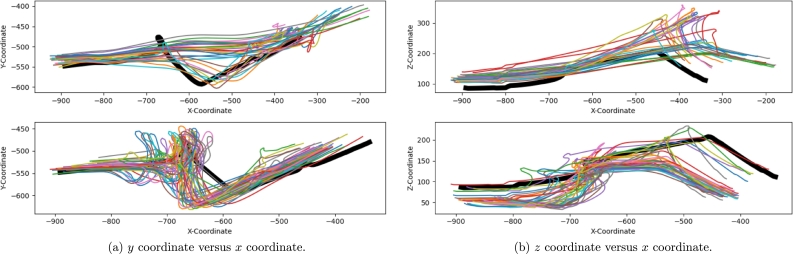
Two clustered deformation modes (up and down); black fat line representing the reference mode.

Fig. 24 shows an important difference in final deformation stage between the two clusters (up and down parts of the figure).

### 5.2 Multi-regression models

**Figure 25:**
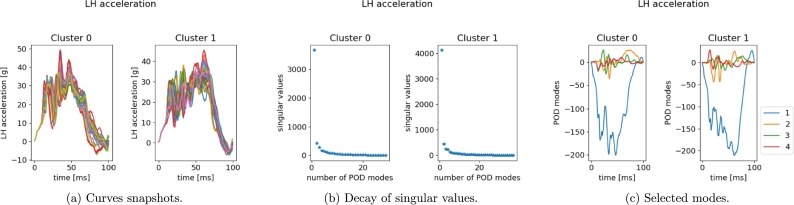
POD results for LH *x*-acceleration snapshots within the two identified clusters.

**Figure 26:**
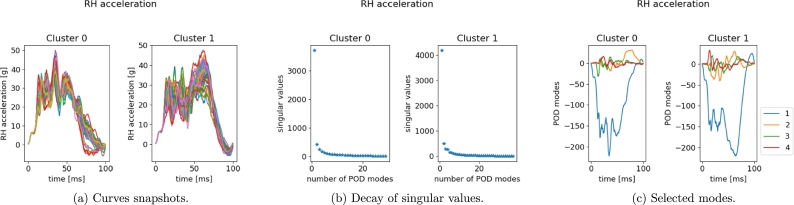
POD results for RH *x*-acceleration snapshots within the two identified clusters.

In Figure 25, Figure 26 we give the results of POD performed separately for each cluster, for left and right measurement points, respectively. As remarked at the end of Section 4, the two clusters identified based on the crash dynamics influence the final part of the acceleration curve (starting from 45 ms). In the first scenario, the measurement node starts decelerating while in the second one it keeps accelerating up to a maximum point (at almost 65 ms) and then suddenly falls. Two sPGD models are thus trained and calibrated, one for each cluster.

### 5.3 Classification

**Figure 27:**
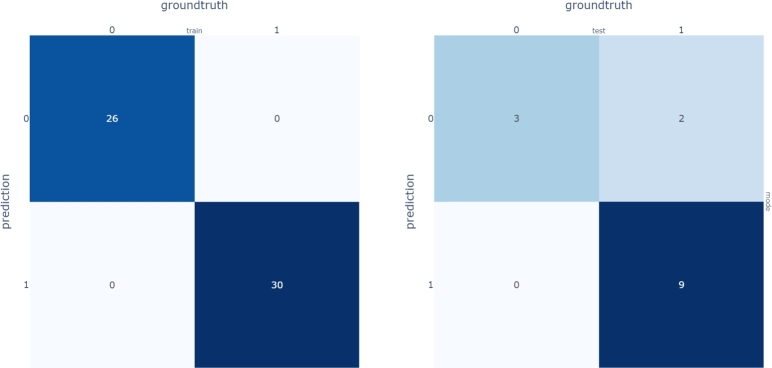
Confusion matrix for Random Forest Classifier.

A classification step shall also be integrated in the procedure since, for a new choice of parameters, one must be able to select the right sub-model for curves prediction. A Random Forest classifier was employed for this study, whose confusion matrix is given in Fig. 27.

**Figure 28:**
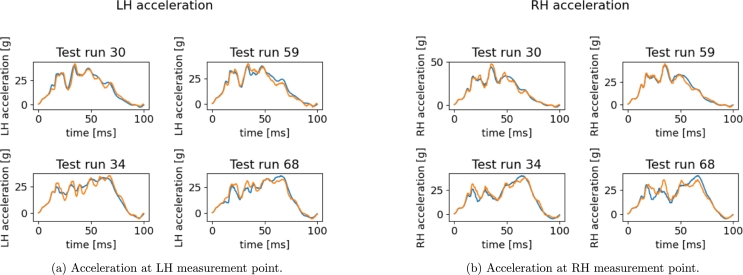
Reconstructed *x*-acceleration using the multi-PGD models (orange: Hi-Fi simulation, blue: Reduced Model).

Finally, in Fig. 28 we give the results on the acceleration predictions. As expected, test runs 30 and 59 belong to the first cluster, while 34 and 68 to the second one.

The first peak and the final part of the curve are now almost perfectly predicted. The average $L^{2}$ norm error is now reduced to 9% on average, against the 15% of the unique model.

## 6 Conclusion, further developments and applications

In the present study a parametric model of the vehicle frontal structure has been built and numerically tested over a full-frontal crash. Thicknesses and material properties of the front side members are parametric.

Virtual plastic materials can be sampled moving near the manifold identified by experimental data collected for the quasi-static material tests. The rate-dependent Krupkowski plasticity curves (i.e., the strain-rate effects) are consequently built according to trained Neural-Network models linking experiments at quasi-static and dynamic tests. The Neural Networks show great accuracy in the prediction from quasi-static to dynamic, meaning that the parametric strain-rate is of great precision.

The model allows to evaluate in real time the influence of 13 parameters (4 thicknesses and 9 material properties), without needs of performing again a full vehicle crash computation. To improve the accuracy of acceleration curves predictions a hierarchical clustering on crash dynamics has been performed and separate multi-regression models have been built. Accordingly, a Random Forest classifier is trained to identify the right cluster for a newly defined run.

Although the accuracy of the model seems to be really satisfactory considering its computational gains, several developments could be attempted for accuracy improvements. First, a reduced parametric space could be considered, being the material parameters range quite wide in this study (from Mild Steel to Press Hardened Steel). Some other strategies, towards specific and physics-informed models, will also be part of our future research in this line. The curves could, indeed, be split in several regimes and separate sub-models could be built for each part of the curve. For this, one could be inspired by the curves alignment methodology proposed in [10]. Such regimes are often evident observing the acceleration-displacement curve and its integral function (RDE as a function of the displacement). Once the different regimes identified, some physical considerations could be embedded in the model to understand which parameters are actively influencing a specific part of the curve. For instance, since in the energy absorption the crash boxes act before then the side members, the parameters of crash boxes shall influence mostly the first part of the pulse. Similarly, the transitory part will mostly be influenced by side members parameters.

Among the applications and advantages of the proposed procedure, there is the monitoring, in real time, of the influence of material parameters on all safety indices like RDE, OLC, first peak acceleration or rebound time. Moreover, defining a parametric metamodel for another crash scenario, e.g. an offset crash, a constrained optimization problem could be considered: find the optimal parameters to minimize the frontal structure weight or manufacturing cost while respecting all crash performance specifications. We may also comment-out about the design space. In this paper, only the frontal structure has been parametrized. However, in our works in progress, we focus also on models where several parts (in the whole vehicle) are parametric and where the reduced-order model is built for all the measured 3D fields. In general, higher the number of parameters, higher the number of required high-fidelity simulations. Although this is a classical limitation of Non-Intrusive ROMs —NI-ROMs—, the sPGD structure allows us to work in the low-data limit even in high-dimensional parametric spaces. The algorithm, as done in this paper, needs sometimes, for the sake of accuracy, to be coupled with fields alignment, clustering and classification. This requires, not only good results from the clustering step, but also the existence of a discriminant in the design space and a classifier able to detect it. From clustering and classification results obtained in [10] and in this work, the multi-sPGD seems being really promising. In our current research we are focusing on developing new methodologies based on a non-intrusive parametric domain-decomposition approach to reduce the computational effort required when many parts (consequently, many parameters) are involved.

## Declarations

### Author contribution statement

Angelo Pasquale, Victor Champaney: Conceived and designed the experiments; Performed the experiments; Analyzed and interpreted the data; Wrote the paper.

Youngtae Kim: Performed the experiments; Analyzed and interpreted the data.

Nicolas Hascoët, Amine Ammar, Francisco Chinesta: Contributed reagents, materials, analysis tools or data.

### Funding statement

This research did not receive any specific grant from funding agencies in the public, commercial, or not-for-profit sectors.

### Data availability statement

The data that has been used is confidential.

### Declaration of interests statement

The authors declare no conflict of interest.

### Additional information

No additional information is available for this paper.

## Footnotes

2 The work is developed in collaboration with Hyundai Motor Company, in the context of the International Research Chair CREATE-ID between ESI Group and ENSAM ParisTech.
